# The PI3K-AKT-mTOR axis persists as a therapeutic dependency in KRAS^G12D^-driven non-small cell lung cancer

**DOI:** 10.1186/s12943-024-02157-x

**Published:** 2024-11-12

**Authors:** W. J. McDaid, L. Wilson, H. Adderley, A. Martinez-Lopez, M. J. Baker, J. Searle, L. Ginn, T. Budden, M. Aldea, A. Marinello, J. V. Aredo, A. Viros, B. Besse, H. A. Wakelee, F. Blackhall, S. Castillo-Lluva, C. R. Lindsay, A. Malliri

**Affiliations:** 1 Division of Cancer Sciences, School of Medical Sciences, Faculty of Biology Medicine and Health, The University of Manchester, Manchester, UK; 2 Cell Signalling Group, Cancer Research UK Manchester Institute, The University of Manchester, Manchester, UK; 3 The Christie NHS Foundation Trust, Manchester, UK; 4 Department of Biochemistry and Molecular Biology, Faculty of Chemical Sciences, Complutense University of Madrid, Madrid, Spain; 5 Skin Cancer and Ageing Group, Cancer Research UK Manchester Institute, The University of Manchester, Manchester, UK; 6 Paris Saclay University, Department of Cancer Medicine, Gustave Roussy Cancer Campus, Villejuif, France; 7 Division of Oncology, Stanford University School of Medicine, Stanford, CA USA; 8 Cancer Research UK Lung Cancer Centre of Excellence, The University of Manchester, Manchester, UK

**Keywords:** KRAS, NSCLC, PI3K-AKT-mTOR pathway, KRAS^G12D^ inhibition

## Abstract

**Introduction:**

KRAS^G12C^ and KRAS^G12D^ inhibitors represent a major translational breakthrough for non-small cell lung cancer (NSCLC) and cancer in general by directly targeting its most mutated oncoprotein. However, resistance to these small molecules has highlighted the need for rational combination partners necessitating a critical understanding of signaling downstream of KRAS mutant isoforms.

**Methods:**

We contrasted tumor development between *Kras*^*G12C*^ and *Kras*^*G12D*^ genetically engineered mouse models (GEMMs). To corroborate findings and determine mutant subtype-specific dependencies, isogenic models of *Kras*^*G12C*^ and *Kras*^*G12D*^ initiation and adaptation were profiled by RNA sequencing. We also employed cell line models of established KRAS mutant NSCLC and determined therapeutic vulnerabilities through pharmacological inhibition. We analysed differences in survival outcomes for patients affected by advanced *KRAS*^*G12C*^ or *KRAS*^*G12D*^-mutant NSCLC.

**Results:**

KRAS^G12D^ exhibited higher potency in vivo, manifesting as more rapid lung tumor formation and reduced survival of KRAS^G12D^ GEMMs compared to KRAS^G12C^. This increased potency, recapitulated in an isogenic initiation model, was associated with enhanced PI3K-AKT-mTOR signaling. However, KRAS^G12C^ oncogenicity and downstream pathway activation were comparable with KRAS^G12D^ at later stages of tumorigenesis in vitro and in vivo, consistent with similar clinical outcomes in patients. Despite this, established KRAS^G12D^ NSCLC models depended more on the PI3K-AKT-mTOR pathway, while KRAS^G12C^ models on the MAPK pathway. Specifically, KRAS^G12D^ inhibition was enhanced by AKT inhibition in vitro and in vivo.

**Conclusions:**

Our data highlight a unique combination treatment vulnerability and suggest that patient selection strategies for combination approaches using direct KRAS inhibitors should be i) contextualised to individual RAS mutants, and ii) tailored to their downstream signaling.

**Supplementary Information:**

The online version contains supplementary material available at 10.1186/s12943-024-02157-x.

## Introduction

Non-small cell lung cancer (NSCLC) is the most common form of lung cancer, diagnosed in up to 85% of patients [1]. It is the leading cause of cancer-related deaths worldwide, with only ~ 20% of NSCLC patients surviving longer than 5 years [2, 3]. Lung adenocarcinoma (LUAD), originating in alveolar type 2 epithelial cells, is the most common histological subtype of NSCLC [4], with KRAS the most frequently mutated oncogenic driver, found in ~ 30% of cases [5]. KRAS gain-of-function mutations are critical for both the initiation and maintenance of tumors [6], with different KRAS point mutations occurring with varying prevalence. KRAS^G12C^ is the most common mutation occurring in up to 40% of KRAS-mutant LUAD, followed by KRAS^G12V^ and KRAS^G12D^, which occur in up to 19% and 15% of KRAS-mutant LUAD, respectively [7].

It is now accepted that NSCLC bearing different KRAS mutations are heterogenous resulting from factors such as varying levels of KRAS activation (GTP-bound KRAS), upregulation of distinct pro-tumorigenic functions and contextual acquisition of secondary mutations exclusive to each KRAS mutation [8–11]. Consequently, a ‘one drug fits all’ approach to targeting KRAS mutant lung cancer is challenging, and treatment should be tailored to the subtype of KRAS mutation. For this to be achieved, it is important to characterise the signaling pathways activated by different KRAS mutants and identify dependencies exclusive to each mutant isoform that can be exploited therapeutically.

In recent years, direct inhibitors of KRAS^G12C^ and KRAS^G12D^ have been developed with KRAS^G12C^ inhibitors, such as sotorasib and adagrasib, now in the clinic, with the former inducing responses in 25–40% of patients [12–16]. However, the efficacy of KRAS^G12C^ inhibitors is limited by several intrinsic resistance mechanisms [17], also expected to impede KRAS^G12D^ inhibitors that are currently being assessed in early-phase clinical trials. In addition, it is now known that KRAS^G12D^ is associated with immune suppression and resistance to PD-L1 therapy compared to other KRAS mutant isoforms [18–20]. It is therefore critical to identify KRAS^G12D^-specific dependencies to target in combination with KRAS^G12D^ inhibition, aiming to improve patient outcomes by overriding anticipated resistance.

In this study, through a comprehensive analysis of isogenic systems with validation in physiologically relevant tumor cell models and NSCLC patient data, we investigated the biological features of KRAS^G12C^ and KRAS^G12D^ mutations and their signaling differences during NSCLC initiation and in established cell models. We identified a KRAS^G12D^-specific mechanism of tumorigenesis which is therapeutically exploitable and potentiates KRAS^G12D^ inhibition.

## Materials and methods

### In vivo studies

All mouse studies were carried out in compliance with UK Home Office regulations with protocols approved by the Cancer Research UK Manchester Institute Animal Welfare and Ethical Review Advisory Body. Generation of the *Kras*^*G12C*^ mouse model, tumor burden, survival and early lesion studies, drug combination studies and histological analyses are described in Supplementary Materials and Methods. The chicken embryo xenograft experiment, which was employed to assess drug combination studies in vivo, is described in Supplementary Materials and Methods. Sequences for gRNA, repair template ultramer and genotyping primers are described in Supplementary Table S1.

### Cell culture

All cell lines were cultured at 37 °C with 5% CO_2_. H358, HCC1171, H1792, H2030, H23, HOP62, A427, SKLU-1 and HCC461 cells were cultured in RPMI-1640 Medium (Gibco, #21,875,034) supplemented with 10% tetracycline-free FBS (Biosera, #FB-1001 T) and 1% penicillin/streptomycin (P/S) (Gibco, #15,140,122). KPAR^G12D^ cells, MEFs and Lenti-X 293 T cells were cultured in DMEM (Gibco, #41,966–029) with 10% tetracycline-free FBS and 1% P/S. MEFs were maintained in 4 µg/mL blasticidin to maintain expression of KRAS transgenes. GEMM-derived tumor cell lines were cultured in DMEM/F12 (Gibco, #11330–032) supplemented with 10% tetracycline-free FBS, 1% P/S, 2 mM glutamine, 1 µM hydrocortisone, 20 ng/mL murine EGF (Cell Signaling, #5331) and 50 ng/mL murine IGF (Bio-techne, #791-MG-050). MLE-12 cells were cultured in DMEM/F12 supplemented with 2% tetracycline-free FBS, 1% P/S, 2 nM glutamine, 5 µg/mL insulin, 10 µg/mL transferrin, 30 nM sodium selenite, 10 nM hydrocortisone, 10 mM HEPES and 10 nM β-estradiol. For experiments, MLE-12 cells were cultured in the same media but with 0.5% tetracycline-free FBS. Lenti-X 293 T cells were purchased from Takarabio. H358, H23, A427, SKLU-1, HCC1171, H1792, H2030, HOP62 and MLE-12 cells were purchased from ATCC. HCC461 cells were kindly donated by John Minna (UT Southwestern, USA). KRAS MEFs were provided by Frederick National Lab for Cancer Research (NCI, USA). KPAR^G12D^ cells were kindly donated by Julian Downward (Crick Institute, UK). Cell lines were regularly authenticated and checked for mycoplasma contamination through in-house facilities. Generation of cell models, proliferation and viability assays, Western blotting, RNA sequencing and analysis, intracellular and surface staining by flow cytometry, caspase-3/-7 detection and propidium iodide staining are described in Supplementary Materials and Methods. Antibodies are documented in Supplementary Table S2.

### Clinical database analysis

575 RAS mutant patients were recruited to the multicentre transatlantic RAS precision medicine (RAS-PM) study from three tertiary cancer centres including The Christie NHS Foundation Trust, The Gustave Roussy Cancer Centre and The Stanford Cancer Institute. Key inclusion criteria were defined as: approval by the ethics committees as required by local or international standards, stage IIIb/IV NSCLC, availability of progression-free survival (PFS) and overall survival (OS) data and confirmed RAS mutant status. Key exclusion criteria included: inconclusive or no confirmed NSCLC diagnosis histologically, patients with cancers wild-type for KRAS and other mutations other than KRAS^G12C^ and KRAS^G12D^, and no PFS or OS data. First-line PFS was defined as time from treatment start, in advanced stage disease, to progression or death from any cause. OS is defined as time from first-line treatment start, in advanced stage disease, to death regardless of cause. Patients still alive at last visit are censored at date of last follow-up. Data collection protocols were approved by local governance committee.

### Statistical analysis

All error bars shown on graphs represent ± standard error of the mean (s.e.m.). The specific statistical tests used are indicated in the figure legends alongside the *p* values and were carried out using GraphPad Prism 10. For comparison between two conditions, statistical tests can be assumed to be a two-tailed Student’s t-test. For multiple comparisons, one-way or two-way ANOVA was used. Log-rank test was used for survival curve analyses. n.s. = not significant, *P < 0.05, **P < 0.01, ***P < 0.001 and **** P < 0.0001.

## Results

### KRAS^G12D^ is more potent than KRAS^G12C^ in driving NSCLC initiation in vivo

**Fig. 1:**
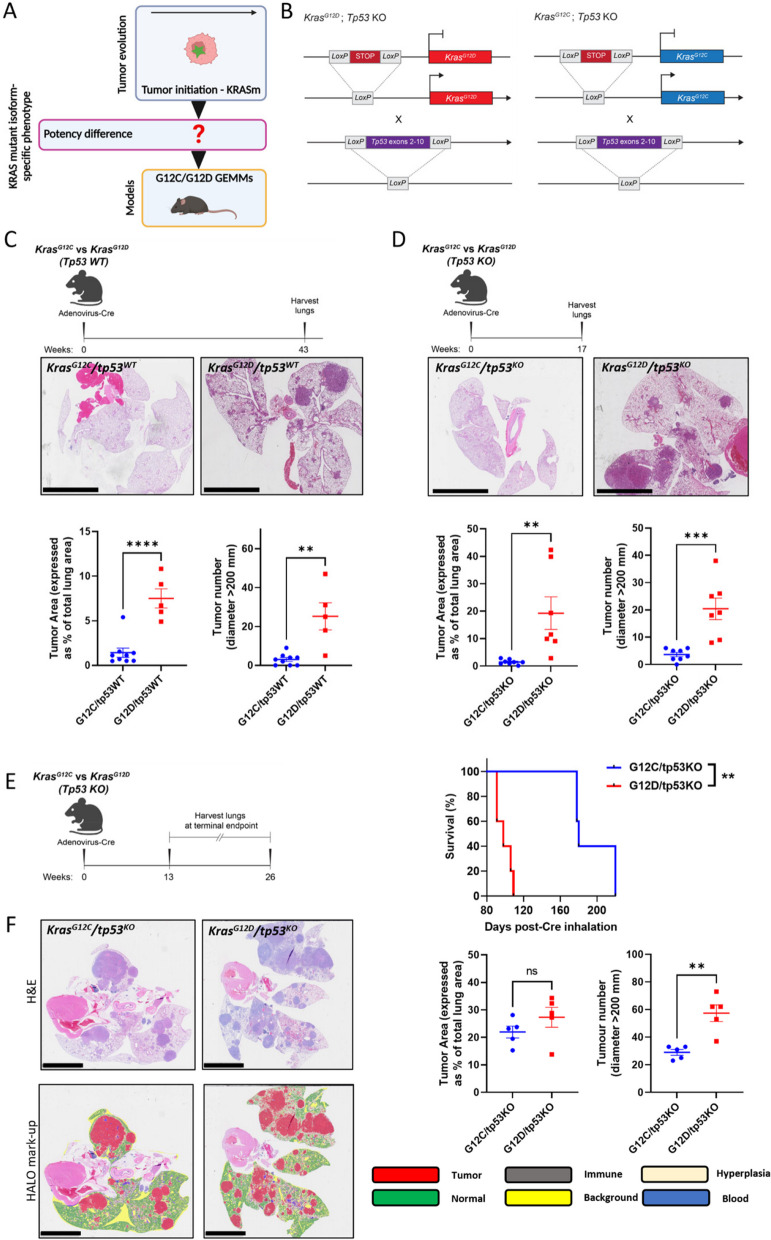
KRAS^G12D^ is more potent than KRAS^G12C^ in driving NSCLC initiation in vivo. **A** Schematic illustrating the use of GEMM models to study the impact of KRAS mutant isoforms on NSCLC initiation. Green star = KRAS mutation. Created with BioRender.com/b49e578. **B** Schematic illustrating KRAS mutant oncogenes silenced by the insertion of a STOP codon flanked by LoxP sites. AdCre administration by inhalation leads to LoxP site recombination, removing the STOP codon allowing KRAS mutant isoform expression in GEMM mice lungs. Conditional KRAS mutant mice were crossed with mice in which *tp53* is also flanked by LoxP sites. AdCre induces LoxP recombination and loss of p53 protein expression. **C** (Above) Timeline of experiment. (Below) Representative H&E sections and HALO quantification of lung tumor area and number per mouse comparing *Kras*^*G12C*^*/tp53*^*WT*^ and *Kras*^*G12D*^*/tp53*^*WT*^ mice 11 months after intranasal AdCre exposure (*n* = 9 *Kras*^*G12C*^*/tp53*^*WT*^ mice and 5 *Kras*^*G12D*^*/tp53*^*WT*^ mice); scale bar = 5 mm. **D** (Above) Timeline of experiment. (Below) Representative H&E sections and HALO quantification of lung tumor area and number comparing *Kras*^*G12C*^*/tp53*^*KO*^ and *Kras*^*G12D*^*/tp53*^*KO*^ mice 4 months after intranasal AdCre exposure (*n* = 7–8 mice per genotype); scale bar = 5 mm. **E** (Left) Timeline of experiment. (Right) Survival analysis for *Kras*^*G12C*^*/tp53*^*KO*^ and *Kras*^*G12D*^*/tp53*^*KO*^ mice after intranasal delivery of AdCre (*n* = 5 mice per genotype, Log-Rank (Mantel-Cox) test). **F** (Top left) Representative H&E images, (Bottom left) HALO mark-up and (Right) HALO quantification of tumor area and number comparing *Kras*^*G12C*^*/tp53*^*KO*^ and *Kras*^*G12D*^*/tp53*^*KO*^ mice from survival study (*n* = 5 mice per genotype); scale bar = 5 mm. **C**, **D** and **F** depict mean ± s.e.m and statistical analysis carried out using unpaired Student’s t-test. ****P < 0.0001, ***P < 0.001, **P < 0.01, ns > 0.05

To examine and compare the oncogenic potency of *Kras*^*G12D*^ and *Kras*^*G12C*^ in vivo, we used a well-characterized genetically-engineered mouse model (GEMM) that harbours latent *Kras*^*G12D*^, whose activation with adenovirus expressing Cre recombinase (AdCre) drives the formation of lung tumors closely resembling human LUAD [21] (Fig. 1A, B). We then used CRISPR/Cas9 technology to convert the aspartic-acid-encoding codon 12 to one encoding cysteine (*Kras*^*G12C*^) to create a *Kras*^*G12C*^ mouse model (Fig. 1B). Thus, we could study relative potency of *Kras*^*G12D*^ and *Kras*^*G12C*^ in a closely controlled in vivo setting. We further combined both *Kras* alleles with conditional *tp53* knockout (*tp53*^*KO*^) to accelerate tumorigenesis and better recapitulate human NSCLC (Fig. 1B) [22].

*Kras*^*G12C*^ mouse models have so far been under-reported in NSCLC research, highlighting the value this tool offers to the investigation of lung cancers driven by this oncogene. We first confirmed that the *Kras*^*G12C*^ mouse model was functional: after intranasal AdCre inhalation, lung tumors were formed which recapitulated tumors resembling lung adenocarcinoma, similarly to the *Kras*^*G12D*^ mouse model (Supplementary Figure S1A). However, when *Kras*^*G12C*^ were compared with *Kras*^*G12D*^ mice matched for time after AdCre inhalation, there was a striking difference in tumorigenic properties between *Kras*^*G12D*^ and *Kras*^*G12C*^ models on both *tp53* wild-type (*tp53*^*WT*^) and *tp53*^*KO*^ backgrounds: *Kras*^*G12D*^-expressing mice had a dramatically increased tumor burden compared to *Kras*^*G12C*^ (Fig. 1C, D). *Kras*^*G12D*^ GEMMs also had increased hyperplasia compared to *Kras*^*G12C*^ GEMMs (Supplementary Figure S1B,S1C). This difference in tumor burden was reflected by shorter median overall survival of *Kras*^*G12D*^*/tp53*^*KO*^ GEMMs (98 days) compared to *Kras*^*G12C*^*/tp53*^*KO*^ GEMMs (180 days) (Fig. 1E). These results were reproduced using an intratracheal method of virus inhalation with similar tumor latency observed (median survival was 211 days for *Kras*^*G12C*^*/tp53*^*KO*^ mice vs 102 days for *Kras*^*G12D*^*/tp53*^*KO*^ mice (Supplementary Figure S1D). However, despite a slight non-significant increase in lung tumor area for *Kras*^*G12D*^*/tp53KO* and comparable hyperplasia areas between *Kras*^*G12C*^*/tp53*^*KO*^ and *Kras*^*G12D*^*/tp53*^*KO*^ in mice euthanized due to disease symptoms (Fig. 1F and Supplementary Figure S1E), *Kras*^*G12D*^ mice had approximately two-fold higher tumor number, constituted mainly by an increased number of smaller tumors (Fig. 1F and Supplementary Figure S1F). These data suggest that *Kras*^*G12D*^ is more effective than *Kras*^*G12C*^ in initiating NSCLC tumors.

### KRAS^G12D^ co-opts the PI3K-AKT-mTOR pathway to promote tumor initiation in NSCLC

**Fig. 2:**
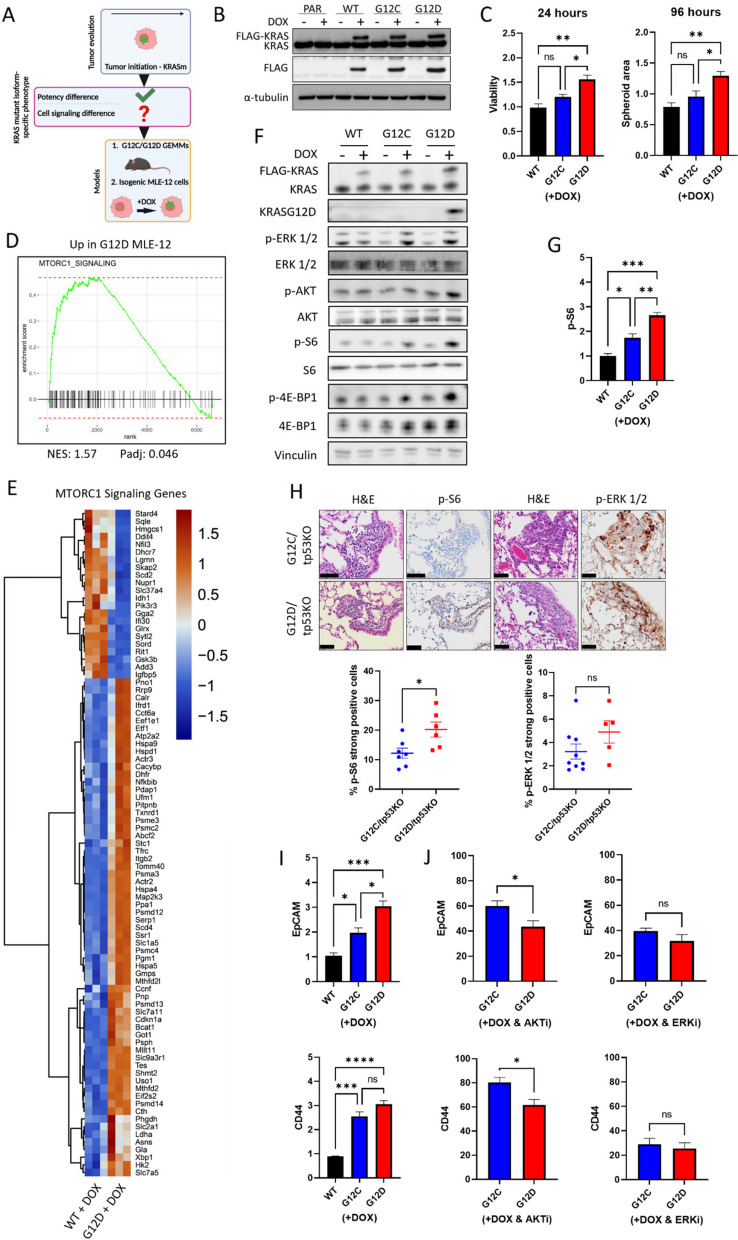
KRAS^G12D^ co-opts the PI3K-AKT-mTOR pathway to promote tumor initiation in NSCLC. **A** Schematic illustrating using GEMM models and isogenic MLE-12 cells to compare the impact of KRAS mutant isoforms on NSCLC initiation and signaling. Green star = KRAS mutation. Created with BioRender.com/b26f425. **B** Western blot analysis of KRAS and FLAG-tagged KRAS upon 24 h exposure of isogenic MLE-12 cells to 100 ng/mL doxycycline. PAR = parental. **C** (Left) MLE-12 spheroid viability upon 24 h 100 ng/mL doxycycline exposure measured by CellTiter-Glo 3D and (Right) MLE-12 spheroid area upon 96 h 100 ng/mL doxycycline exposure measured by ImageJ. Data normalised to untreated (no doxycycline) control (*n* = 3 at 24 h and *n* = 4 at 96-h). **D** GSEA showing that mTORC1 signaling genes are positively correlated with KRAS^G12D^ expression compared to KRAS^WT^. **E** Heatmap showing DEGs belonging to mTORC1 signaling gene-set when comparing *KRAS*^*G12D*^ to *KRAS*^*WT*^ MLE-12 spheroids 24 h after 100 ng/mL doxycycline exposure (*n* = 3). **F** Western blot analysis of ERK, AKT, S6 and 4E-BP1 phosphorylation (Ser65) 24 h after exposure of isogenic MLE-12 spheroids to 100 ng/mL doxycycline. Representative of 3 independent experiments. **G** Flow cytometric quantification of S6 phosphorylation levels upon 24 h exposure of isogenic MLE-12 spheroids to 100 ng/mL doxycycline. Data normalised to untreated (no doxycycline) control (*n* = 3). **H** (Above) Representative immunohistochemical staining and (Below) quantification of ERK and S6 phosphorylation in early lung lesions of *Kras*^*G12C*^*/tp53*^*KO*^ and *Kras*^*G12D*^*/tp53*^*KO*^ mice (*n* = 6–9 mice per genotype); scale bar = 50 µm. **I** Flow cytometric quantification of surface CD44 and EpCAM in isogenic MLE-12 spheroids upon 24 h exposure to 100 ng/mL doxycycline. Data normalised to untreated (no doxycycline) control (*n* = 3). **J** Flow cytometric quantification of surface CD44 and EpCAM in isogenic MLE-12 spheroids upon 24 h exposure to 100 ng/mL doxycycline in the presence of (Left) 1 µM capivasertib (AKTi) or (Right) 1 µM ulixertinib (ERKi). Data normalised to DMSO control (*n* = 4). **C**, **G**, **I** and **J** depict mean ± s.e.m and statistical analysis carried out using one-way ANOVA test. (H) depicts mean ± s.e.m and statistical analysis carried out using unpaired Student’s t-test. ****P < 0.0001, ***P < 0.001, **P < 0.01, *P < 0.05, ns > 0.05. DOX = doxycycline

We next asked whether the increased oncopotency of *Kras*^*G12D*^ compared to *Kras*^*G12C*^ may be due to signaling differences immediately downstream upon tumor initiation. To investigate this, we generated an isogenic KRAS mutant initiation model using an immortalised murine lung alveolar type 2 cell line, MLE-12, that is non-tumorigenic when inoculated in mice [23], and modified it to ectopically express either flag-tagged wildtype KRAS (KRAS^WT^), KRAS^G12C^ or KRAS^G12D^ under the control of a doxycycline-regulated promoter (Fig. 2A, B). We confirmed the correct expression of each isoform through exposure of the isogenic panel to doxycycline in the presence or absence of the KRAS^G12C^ inhibitor (G12Ci) sotorasib. Exposure to G12Ci only affected *Kras*^*G12C*^ MLE-12 cells by binding to KRAS and switching off MAPK signaling as evidenced by reduced phosphorylated ERK. Additionally, we detected KRAS^G12D^ protein expression using a KRAS^G12D^-specific antibody only in *Kras*^*G12D*^ MLE-12 cells (Supplementary Figure S2A). MLE-12 cells were next cultured in ultra-low attachment (ULA) plates to induce growth as 3D spheroids, mimicking more closely the physical characteristics of cancer cells in a tumor [24]. Upon doxycycline treatment, an increase in metabolic activity and spheroid size was observed in *Kras*^*G12D*^- compared to *Kras*^*G12C*^-initiated cells, both features indicative of increased viability and proliferation respectively, consistent with our in vivo data (Fig. 2C and Supplementary Figure S2B).

We next asked what could be driving the increased proliferation of *Kras*^*G12D*^-initiated cells. Gene expression profiling of the isogenic MLE-12 panel comparing either *Kras*^*G12D*^ or *Kras*^*G12C*^ to *Kras*^*WT*^ 24 h after doxycycline exposure revealed that *Kras*^*G12D*^ had a stronger impact on the transcriptome with 6664 differentially expressed genes (DEGs) compared to *Kras*^*WT*^, whereas 4566 genes were altered between *Kras*^*G12C*^ and *Kras*^*WT*^ (Supplementary Table S3). From these gene changes, we identified mTORC1 signaling as a significantly enriched gene-set when comparing *Kras*^*G12D*^ to *Kras*^*WT*^ (Fig. 2D, E), not seen when comparing *Kras*^*G12C*^ to *Kras*^*WT*^, suggesting higher activation of this pathway in cells initiated with *Kras*^*G12D*^. Alternatively, by comparing each isogenic cell line after addition of doxycycline to its untreated counterpart, there was a strikingly higher number of differentially expressed genes belonging to the mTORC1 signaling gene-set in cells with *Kras*^*G12D*^ expression compared to cells with *Kras*^*WT*^ or *Kras*^*G12C*^ (Supplementary Figure S2C). Taken together, *Kras*^*G12D*^ expression leads to a more extensive transcriptional reprogramming, upregulating several mTORC1-associated genes compared to *Kras*^*G12C*^ or *Kras*^*WT*^ MLE-12 cells.

As mTORC1 is part of the PI3K-AKT-mTOR axis, a key effector pathway of RAS signaling [25], we postulated that KRAS^G12D^ co-opts the PI3K-AKT-mTOR pathway to a higher extent than KRAS^G12C^ and aimed to explore this pathway further as a potential mechanism underpinning the greater potency of KRAS^G12D^. Firstly, we analysed if the PI3K-AKT-mTOR pathway was hyperactivated in KRAS^G12D^-initiated cells. Indeed, higher phosphorylation of the mTORC1 activator, AKT, and the mTORC1 substrates ribosomal S6 and 4E-BP1, were evident in *Kras*^*G12D*^ MLE-12 cells 24 h after exposure to doxycycline, consistent with our gene expression data (Fig. 2F, G). Next, using ERK phosphorylation as a measure of MAPK signaling, we observed no difference in MAPK signaling between the two mutant isoforms (Fig. 2F). By further analysing our gene expression data, we saw that DEGs related to MAPK signaling were similar between mutant isoforms (Supplementary Figure S2D). Treatment of parental MLE-12 cells with doxycycline confirmed that doxycycline does not affect either of these pathways (Supplementary Figure S2E), nor expression of exogenous KRAS^WT^ to levels matching mutant KRAS (Fig. 2F). Higher S6 phosphorylation was also noted in early *Kras*^*G12D*^ lung lesions in vivo compared to *Kras*^*G12C*^ lesions, whilst there was no significant difference in the level of ERK phosphorylation between the two mutant isoforms (Fig. 2H). Finally, we selected CD44 and EpCAM as two markers of NSCLC initiation [26–28] and showed that their expression is increased upon induction of the mutant isoforms only (Fig. 2I). Inhibition of AKT using an FDA-approved AKT inhibitor (capivasertib) [29] reduced expression of these markers to a greater extent in *Kras*^*G12D*^ MLE-12 cells, whereas inhibition of ERK using ulixertinib reduced their expression to comparable levels in both cell lines (Fig. 2J). This suggests that the two mutant isoforms require input from the MAPK pathway for tumor initiation to the same extent, whereas the PI3K-AKT-mTOR pathway is required more during *Kras*^*G12D*^-driven initiation. Overall, these data confirmed our gene expression data and highlighted a possible role for the PI3K-AKT-mTOR pathway in tumor initiation by *Kras*^*G12D*^.

### Long-term KRAS^G12D^-exposed cells display specific PI3K-AKT-mTOR pathway dependency

**Fig. 3:**
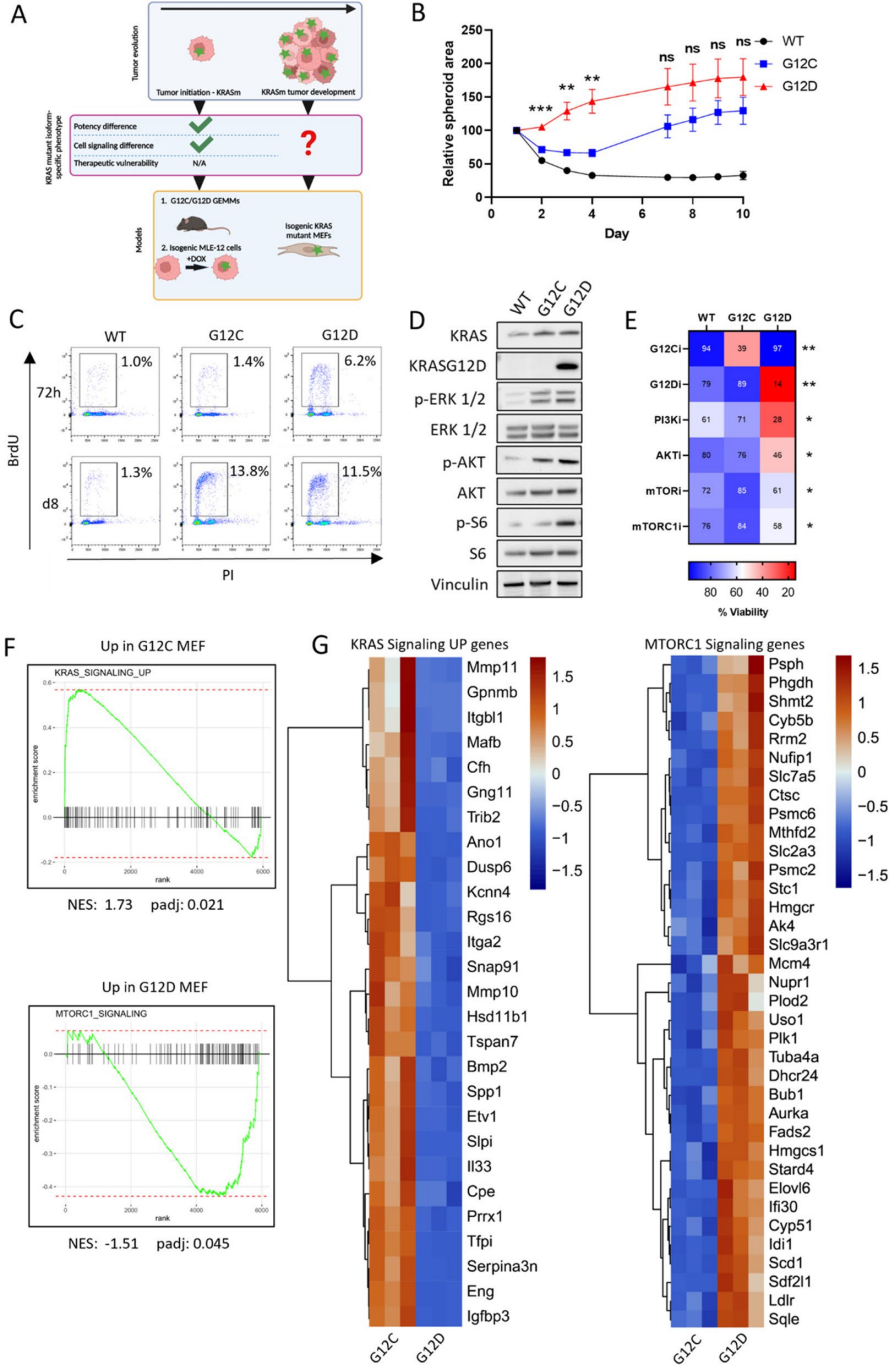
Long-term KRAS^G12D^-exposed cells display specific PI3K-AKT-mTOR pathway dependency. **A** Schematic illustrating using isogenic KRAS MEFs to determine growth rates, signaling differences and therapeutic vulnerabilities conferred by long-term expression of KRAS mutant isoforms. Green star = KRAS mutation. Created with BioRender.com/b26f425. **B** Spheroid area of isogenic KRAS MEFs quantified using ImageJ. Spheroid areas over time were normalised to spheroid area at day 1 (*n* = 3). Mean ± s.e.m. depicted. **C** BrdU/PI staining of isogenic KRAS MEFs at 72 h and 8 days (d8) in 3D. Proliferating cells are expressed as % BrdU-positive. Representative of three independent experiments. **D** Western blot analysis of ERK, AKT and S6 phosphorylation levels in isogenic KRAS MEFs at 24 h in 3D. Representative of three independent experiments. **E** Viability of isogenic KRAS MEFs in response to 100 nM sotorasib (G12Ci), 100 nM MRTX1133 (G12Di), 1 µM buparlisib (PI3Ki), 10 µM capivasertib (AKTi), 1 µM everolimus (mTORi) and rapamycin (mTORC1i) in 3D. Viability was measured after 72 h of drug exposure by CellTiter-Glo 3D. Viability expressed as % of DMSO control (*n* = 4). Mean depicted. **F** GSEA showing that mTORC1 signaling genes are positively correlated with KRAS^G12D^ expression and KRAS Signaling Up genes are positively correlated with KRAS^G12C^ expression. **G** Heatmaps showing DEGs belonging to mTORC1 Signaling and KRAS Signaling UP gene-sets comparing *Kras*^*G12C*^ to *Kras*^*G12D*^ MEFs (*n* = 3 per genotype). Statistical significance analysed using one-way ANOVA (between each time point for B or between drug treatment for E) but only significance between *Kras*^*G12C*^ and *Kras*^*G12D*^ MEFs presented. ***P < 0.001, **P < 0.01, *P < 0.05, ns > 0.05

Having established the allele-specific role of *Kras*^*G12D*^ in facilitating tumor initiation and uncovering its increased activation of the PI3K-AKT-mTOR pathway compared to *Kras*^*G12C*^, we next sought to explore differences between KRAS^G12C^ and KRAS^G12D^ in advanced disease to determine if growth characteristics and signaling differences are maintained during tumor evolution (Fig. 3A). As KRAS-mutant NSCLC tumors are extremely heterogenous, largely due to the diversity of their genetic alterations [9], comparing KRAS-mutant phenotypes using patient samples or NSCLC cell lines is challenging. Therefore, we began by using a panel of isogenic MEFs, initially engineered to become ‘Ras-less’ [30] and further genetically modified to express either KRAS^WT^, KRAS^G12C^ or KRAS^G12D^ [31]. Therefore, we could compare KRAS mutant isoforms directly without the confounding effects of co-mutations seen in lung tumors. As these cells have been cultured long-term in the presence of KRAS mutant alleles, we considered that KRAS mutant isoform-dependent adaptations have developed that may reflect features of established tumors.

First, we observed that *Kras*^*WT*^ or *Kras*^*G12C*^ MEFs were unable to proliferate when cultured in agarose, depriving cells of their anchorage. However, *Kras*^*G12D*^ MEFs proliferated and formed colonies (Supplementary Figure S3A). To determine growth over time, we cultured the MEFs as spheroids in ULA plates and observed that *Kras*^*G12D*^ MEFs were again able to proliferate. In contrast, *Kras*^*WT*^ and *Kras*^*G12C*^ MEFS were initially unable to proliferate, but over time *Kras*^*G12C*^ MEFs began proliferating at the same rate as *Kras*^*G12D*^ MEFs (Fig. 3B, C and Supplementary Figure S3B). Similar to MLE-12 cells, ERK phosphorylation levels were comparable between *Kras*^*G12C*^ and *Kras*^*G12D*^ MEFs after 24 h of culturing in 3D, while AKT and S6 phosphorylation were higher in *Kras*^*G12D*^ MEFs (Fig. 3D and Supplementary Figure S3C), suggesting that *Kras*^*G12D*^-specific PI3K-AKT-mTOR hyperactivation persists beyond initiation. To test if the PI3K-AKT-mTOR pathway supported anchorage-independent growth of *Kras*^*G12D*^ MEFs, we inhibited different nodes in the pathway, such as PI3K with buparlisib, AKT with capivasertib and either mTOR or mTOR complex 1 (mTORC1) with FDA-approved everolimus or rapamycin [32], respectively, and observed that *Kras*^*G12D*^ MEFs were more sensitive than *Kras*^*WT*^ or *Kras*^*G12C*^ MEFs (Fig. 3E).

We next carried out gene expression profiling of *Kras*^*G12C*^ and *Kras*^*G12D*^ MEFs on day 8, aiming to determine differential signaling when both *Kras*^*G12C*^ and *Kras*^*G12D*^ MEFs were proliferating at the same rate. We observed that *Kras*^*G12C*^ MEFs had increased gene expression associated with KRAS signaling (KRAS Signaling UP) whilst, similarly to *Kras*^*G12D*^ MLE-12 cells, *Kras*^*G12D*^ MEFs had increased expression of MTORC1 signaling genes (Fig. 3F, G). Collectively, these data suggest that, in cells exposed long-term to KRAS mutant isoforms, proliferation differences may become less apparent over time, however signaling differences persist as indicated by the gene expression profiles. In order to increase oncogenicity and proliferate, KRAS^G12C^ hyperactivates KRAS signaling which may be relevant to KRAS^G12C^-driven tumor evolution. In contrast, KRAS^G12D^ relies more on PI3K-AKT-mTOR signaling, even beyond initiation, which may be therapeutically exploitable.

### KRAS^G12C^ and KRAS^G12D^ NSCLC cell lines exhibit RAS effector-specific dependencies

**Fig. 4:**
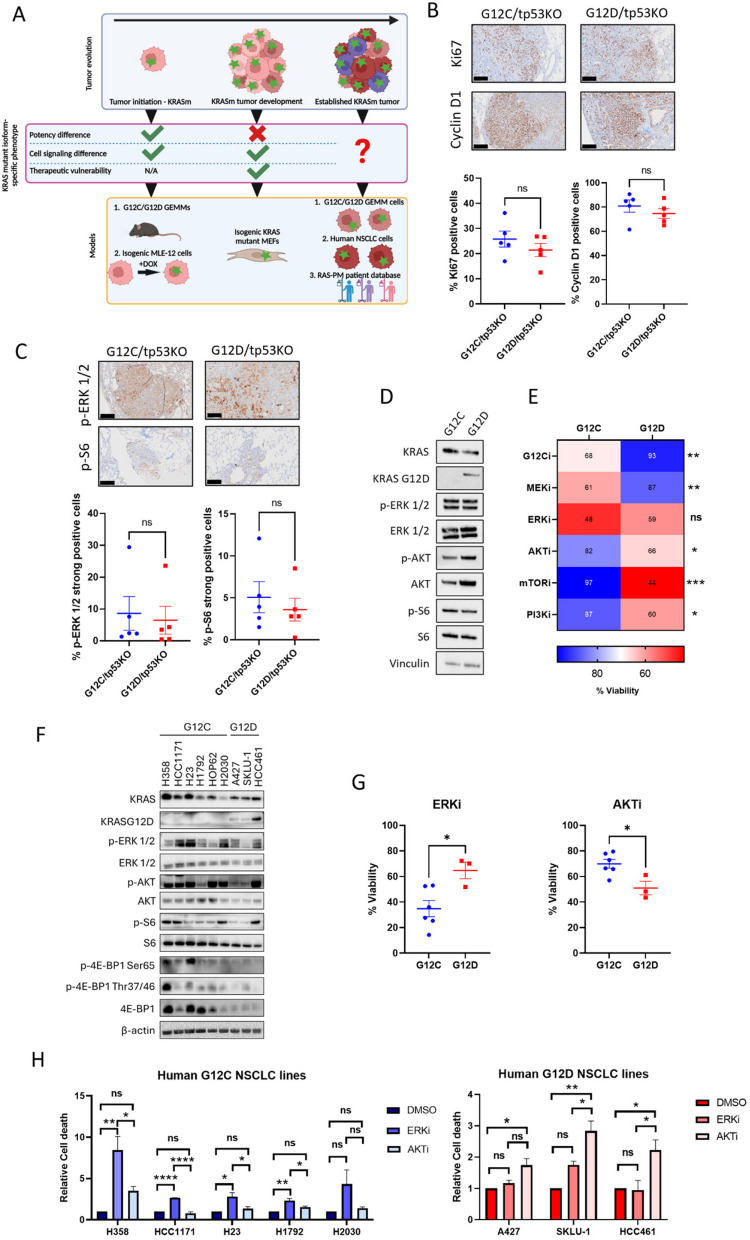
KRAS^G12C^ and KRAS^G12D^ NSCLC cells exhibit RAS effector-specific dependencies. **A** Schematic illustrating using GEMM-derived NSCLC cell lines, human NSCLC cell lines and patient data to determine growth rates, signaling differences and therapeutic vulnerabilities imposed by KRAS mutant isoforms in advanced disease. Green star = KRAS mutation. Created with BioRender.com/t58e004. **B** (Above) Representative immunohistochemical staining and (Below) quantification of Ki67 and Cyclin D1 expression in established lung tumors of *Kras*^*G12C*^*/tp53*^*KO*^ and *Kras*^*G12D*^*/tp53*^*KO*^ mice (*n* = 5 per genotype). Scale bar = 200 µm. **C** (Above) Representative immunohistochemical staining and (Below) quantification of ERK and S6 phosphorylation in established lung tumors of *Kras*^*G12C*^*/tp53*^*KO*^ and *Kras*^*G12D*^*/tp53*^*KO*^ mice (*n* = 5 per genotype). Scale bar = 200 µm. **D** Western blot analysis of ERK, AKT and S6 phosphorylation in *Kras*^*G12C*^ and *Kras*^*G12D*^ mTCLs at 48 h in 3D. Representative of three independent experiments. **E** Viability of *Kras*^*G12C*^ and *Kras*^*G12D*^ mTCLs in response to 1 µM sotorasib (G12Ci), 10 µM U0126 (MEKi), 10 µM ulixertinib (ERKi), 1 µM buparlisib (PI3Ki), 10 µM capivasertib (AKTi) and 10 nM everolimus (mTORi). Viability was measured after 48 h of drug exposure by crystal violet staining. Viability expressed as % of DMSO control (*n* = 3). **F** Western blot analysis of ERK, AKT, S6 and 4E-BP1 phosphorylation of *Kras*^*G12C*^ and *Kras*^*G12D*^ human NSCLC cell lines 48 h in 3D. Representative of three independent experiments. **G** Viability of human *KRAS*^*G12C*^ and *KRAS*^*G12D*^ NSCLC cell lines in response to 10 µM ulixertinib (ERKi) and 10 µM capivasertib (AKTi) in 3D. Viability was measured after 72 h of drug exposure by CellTiter-Glo 3D. Viability expressed as % of DMSO control (*n* = 3). **H** Cell death analyses of human (Left) *KRAS*^*G12C*^ and (Right) *KRAS*.^*G12D*^ NSCLC cell lines in response to 10 µM ulixertinib (ERKi) and 10 µM capivasertib (AKTi) in 3D. Cell death was measured after 48 h of drug exposure by flow cytometric quantification of PI staining. Data normalised to DMSO control (*n* = 3). **B**, **C**, **E** and **G** depict mean ± s.e.m and statistical analysis carried out using unpaired Student’s t-test. (H) depicts mean ± s.e.m and statistical analysis carried out using one-way ANOVA ****P < 0.0001, ***P < 0.001, **P < 0.01, *P < 0.05, ns > 0.05

Our above findings from the mutant KRAS MEFs led us to hypothesise that, once KRAS^G12C^- and KRAS^G12D^-driven tumors are established, the difference in potency between the two variants becomes less evident. Given that precision medicine and KRAS inhibitors are usually administered in the context of stage IV NSCLC, we asked whether KRAS mutant-specific dependency on the pathways described above persists in established NSCLC tumors and cell lines, conferring isoform-specific vulnerabilities (Fig. 4A). First, we carried out immunohistochemical (IHC) staining of tumors from the GEMM survival study (Fig. 1E) for markers of proliferation (Ki67), cell cycle progression (Cyclin D1) and ERK and S6 activation. Interestingly, we did not observe significant differences in the levels of staining for these proteins between *Kras*^*G12C*^*/tp53*^*KO*^ and *Kras*^*G12D*^*/tp53*^*KO*^ lung tumors (Fig. 4B, C) implying that there is no longer a potency (proliferation) or signaling difference between the two mutant isoforms, possibly due to acquired mutations affecting activation of these pathways [33]. In agreement, there was no significant difference in proliferation (Supplementary Figure S4) or ERK, AKT and S6 activation (Fig. 4D) between murine tumor cell lines (mTCL) derived from *Kras*^*G12C*^*/tp53*^*KO*^ and *Kras*^*G12D*^*/tp53*^*KO*^ GEMMs, further implying that the genotype-specific difference in potency and signaling was lost in established tumors. However, *Kras*^*G12C*^ mTCL was more sensitive to ERK and MEK inhibition, whilst *Kras*^*G12D*^ mTCL was more sensitive to PI3K, AKT and mTOR inhibition (Fig. 4E), despite both cell lines showing similar levels of MAPK and PI3K-AKT-mTOR pathway activation, implying that the vulnerabilities persist independently of phosphorylation levels.

To determine if these findings were also relevant to human NSCLC, we first examined differences in KRAS mutant isoform-specific survival outcomes from an internationally recruited cohort of advanced KRAS-mutant NSCLC patients, the RAS-Precision Medicine (RAS-PM) database. Of 575 patients recruited, 240 were affected by cancers harboring *KRAS*^*G12C*^, compared to 92 patients with cancers harboring *KRAS*^*G12D*^ (Supplementary Figure S5A). There were no significant differences in baseline characteristics between *KRAS*^*G12C*^ and *KRAS*^*G12D*^ mutant patients (Supplementary Table S4). There was also no significant difference in either 1st line progression-free survival (PFS) or overall survival (OS) (Supplementary Figure S5B,5C). To test whether mutant-subtype specific differences were more apparent at earlier stages of tumorigenesis in a clinical cohort, we next extracted data from cBioPortal to examine relative differences between KRAS^G12C^ and KRAS^G12D^ NSCLC (Supplementary Figure S5D) [34, 35]. We analyzed data from three cohorts: NSCLC TRACERx study (2017) [36], TCGA Firehose Legacy and Pan-Lung Cancer study (2016) [37]. In contrast to RAS-PM, this cohort was considered early stage and operable, with the intention of examining differences in RAS subtypes at point of diagnosis rather than deterioration. In line with our preclinical observations, the proportion of KRAS^G12D^ T3 and T4 stage tumors was higher compared to KRAS^G12C^ NSCLC (Supplementary Figure S5E). Taken together, these clinical results parallel our in vitro and in vivo findings, highlighting that the tumorigenic strength of KRAS^G12D^ becomes less apparent at late stages of NSCLC, whereby the oncopotency of KRAS^G12C^ appears to ‘catch up’ with KRAS^G12D^.

Despite the loss of potency, we wondered if signaling and therapeutic differences persist in advanced human disease. We selected a panel of 6 KRAS^G12C^ and 3 KRAS^G12D^ human NSCLC cell lines. We first assessed proliferation rates among these lines and saw no significant difference in proliferation, underpinning the late-stage in vivo and patient data (Supplementary Figure S6A). Similar to established tumours in GEMMs, there was no clear differences in ERK, AKT and S6 activation between human KRAS^G12C^ and KRAS^G12D^ cell lines (Fig. 4F), again likely due to factors such as genomic heterogeneity between cell lines or Epithelial-Mesenchymal Transition influencing pathway activation [38]. Unexpectedly and contrary to the isogenic initiation model in which we observed KRAS^G12D^-specific hyperphosphorylation of protein translation repressor 4E-BP1, we saw KRAS^G12D^-specific downregulation of total 4E-BP1 (Fig. 4F). This ultimately has the same implication as 4E-BP1 hyperphosphorylation which is to promote protein translation [39]. We speculate that this may have developed during KRAS^G12D^-driven tumor progression and supports the relationship between KRAS^G12D^ and mTORC1 signaling. We then mined publicly available data from the Cancer Cell Line Encyclopedia (CCLE) and compared gene expression data between *KRAS*^*G12C*^ and *KRAS*^*G12D*^ cell lines [40]. Interestingly, human *KRAS*^*G12C*^ lines were enriched for genes related to increased KRAS signaling, while human *KRAS*^*G12D*^ lines were enriched for genes related to PI3K-AKT-mTOR and specifically mTORC1 signaling (Supplementary Figure S6B), similarly to our MEF gene expression data. Subsequently, we tested the impact of MEK, ERK and AKT inhibition in these NSCLC cell lines and observed that *KRAS*^*G12C*^ lines were more sensitive to ERK or MEK inhibition, while *KRAS*^*G12D*^ lines were more sensitive to AKT inhibition (Fig. 4G and Supplementary Figure S6C). Additionally, we observed higher cell death after ERK or AKT inhibition in *KRAS*^*G12C*^ or *KRAS*^*G12D*^ cells, respectively (Fig. 4H). Altogether, these data imply that, in advanced disease, the difference in potency between these two KRAS mutant isoforms is no longer apparent in terms of proliferation and immediate signaling. However, *KRAS*^*G12C*^ and *KRAS*^*G12D*^ cells are more susceptible to MAPK and PI3K-AKT-mTOR pathway inhibition, respectively. Therefore, these mutant-subtype specific treatment vulnerabilities persist despite the loss of clear differences in oncogenic signaling and phenotype at this point of NSCLC evolution.

**Fig. 5:**
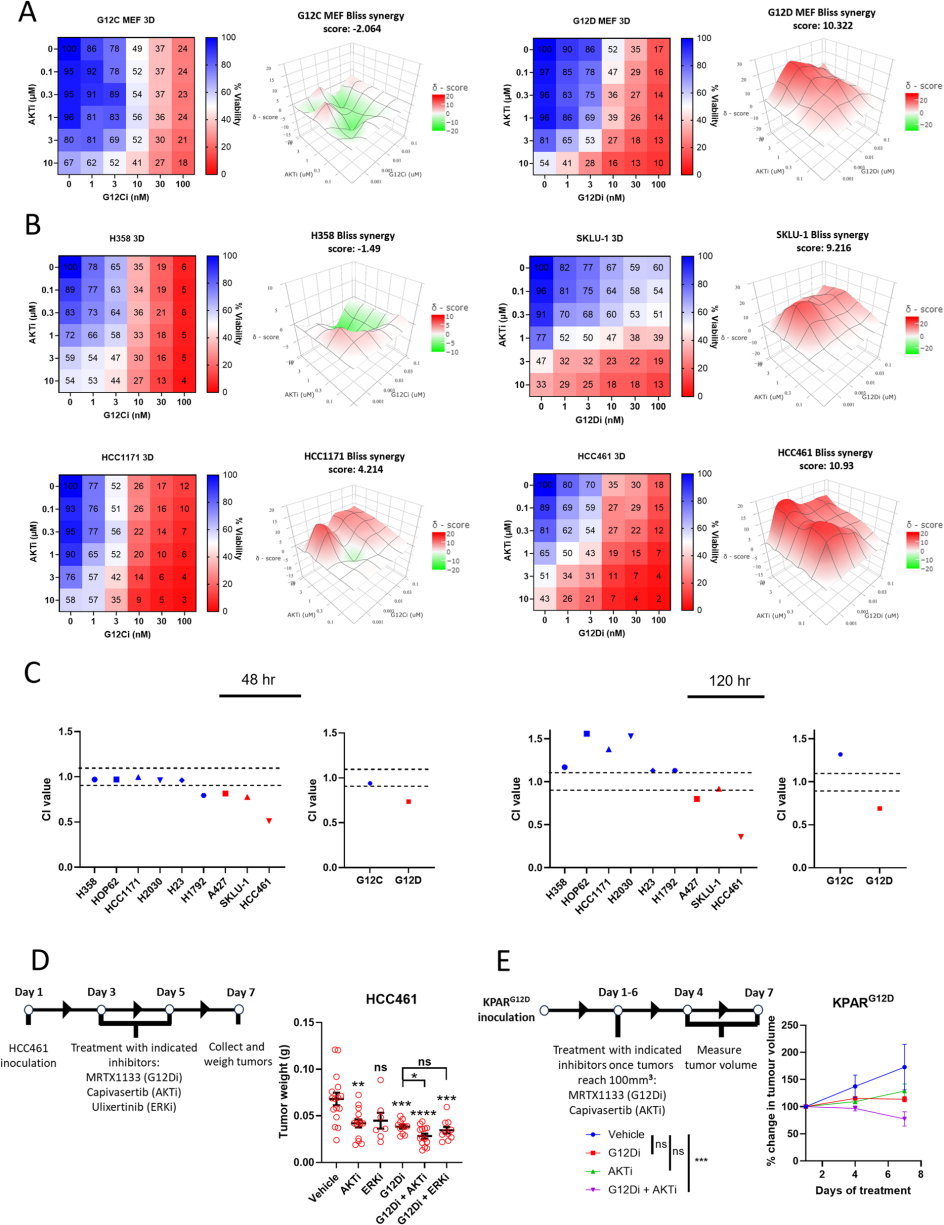
KRAS^G12D^ inhibition and PI3K-AKT-mTOR inhibition synergise in KRAS^G12D^ cells. **A** Isogenic MEFs were treated with increasing concentrations of either sotorasib (G12Ci) or MRTX1133 (G12Di) and capivasertib (AKTi) in 3D. 72 h later, viability was measured by CellTiter-Glo 3D. Viability expressed as % of DMSO control. Drug interaction was calculated using Bliss synergy scoring (*n* = 3). **B** The same conditions and analysis as in 5A in H358 and HCC1171 (KRAS^G12C^) and SKLU-1 and HCC461 (KRAS^G12D^) NSCLC cell lines (*n* = 3). **C** Human NSCLCs were treated with either 1 nM (for H358, HOP62, H2030 and HCC1171), 5 nM (H1792) or 10 nM (H23) sotorasib (G12Ci) or 10 nM MRTX1133 (G12Di) (for A427, SKLU-1 and HCC461) or 10 µM capivasertib (AKTi) and a combination of both in 3D. 48 and 120 h later, viability was measured by CellTiter-Glo 3D. CI values were calculated and presented per cell line and as a mean of all cell lines per genotype (*n* = 3). **D** (Left) Timeline of experiment. (Right) Tumor weights after HCC461 cells were inoculated onto the chorioallantoic membrane of 10-day old chicken embryos and exposed to two rounds of treatment with 5 nM MRTX1133 (G12Di), 50 μM capivasertib (AKTi) or 5 μM ulixertinib (ERKi) individually or as part of combination treatments. (*n* = 7–16 tumors per condition). g = grams. **E** (Left) Timeline of experiment. (Right) Relative tumor volumes of KPAR^G12D^ tumors after inoculation onto the flanks of C57BL/6 mice which were exposed to 6 daily treatments of 10 mg/kg MRTX1133 (G12Di) or 100 mg/kg capivasertib (AKTi) individually or in combination. Tumors represented as % change in volume relative to day 1 measurements (*n* = 3–4 tumors per condition). D depicts mean ± s.e.m and statistical analysis carried out using a one-way ANOVA with Tukey’s post-test and a t-test for comparisons between G12Di and the combination treatment. **E** depicts mean ± s.e.m and statistical analysis carried out using a two-way ANOVA with Tukey’s multiple comparison’s test. ****P < 0.0001, ***P < 0.001, **P < 0.01, **P < 0.05, ns > 0.05. Note: CI = combination index. For CI analysis, points appearing above the top dotted line signify drug antagonism. Points appearing between the top and bottom dotted line signify drug additivity. Points appearing below the bottom dotted line signify drug synergism. For bliss synergy scoring (BSS), a value of less than -10 signifies drug antagonism, a value of between -10 and 10 signifies drug additivity and a value of above 10 signifies drug synergy

The clinical development of direct KRAS^G12C^ inhibitors provides our most advanced current means of inhibiting KRAS [12]. However, the clinical efficacy of KRAS^G12C^ inhibition (G12Ci) in NSCLC is hindered by intrinsic factors such as pathway re-activation and feedback/bypass pathways which often result in resistance [17, 41]. It is expected that resistance will circumvent KRAS^G12D^ inhibition (G12Di) in NSCLC, with reports of resistance mechanisms and combination strategies already emerging in colorectal cancer [42, 43]. Thus, it is vital to explore potential combination therapies to minimise resistance and maximise the potential of G12Di. Having identified the PI3K-AKT-mTOR axis as a KRAS^G12D^-specific vulnerability, we next examined whether its inhibition combines effectively with G12Di in NSCLC. For this, we used two methods of calculating drug interaction – (i) the co-efficient of drug interaction (CDI) Eq. [44] and (ii) Bliss Synergy scoring (BSS) with SynergyFinder [45]. We also directly compared these results with the combined effect of G12Ci and AKTi to inform us whether this is a KRAS^G12D^-specific drug combination effect. Firstly, using our isogenic MEF panel and our GEMM-derived cell lines of KRAS^G12C^ and KRAS^G12D^-driven NSCLC, we saw that the combined effect of G12Di and AKTi was synergistic and more potent (CDI < 0.9) compared to G12Ci and AKTi in both models (Supplementary Figure S7A and B). Moreover, through conducting dose response matrices of increasing concentrations of G12Di and AKTi, we found that in our isogenic MEF panel the combined effect of G12Di + AKTi was again synergistic (BSS of 10.322) and, overall, more potent compared to that of the G12Ci + AKTi combination, where an additive effect was observed (BSS of -2.064) (Fig. 5A).

We next exposed two human KRAS^G12C^ NSCLC cell lines (H358 and HCC1171) and two KRAS^G12D^ NSCLC cell lines (HCC461 and SKLU-1) to increasing doses of G12Ci or G12Di and AKTi and again saw that the combination of G12Di + AKTi was either highly additive (SKLU-1 – BSS of 9.216) or synergistic (HCC461 – BSS of 10.93) (Fig. 5B). Interestingly, SKLU-1 cells are more resistant to G12Di than HCC461 cells as evidenced by the monotherapy responses but both cell lines have similar BSS, implying that co-targeting AKT is effective in both G12Di sensitive and relatively resistant settings. The combination of G12Ci + AKTi was either additive (HCC1171 – BSS of 4.214) or weakly additive (H358 – BSS of -1.49) in KRAS^G12C^ cell lines (Fig. 5B). Next, we utilised our full panel of human KRAS^G12C^ and KRAS^G12D^ cell lines, exposing them to either a combination of G12Ci + AKTi or G12Di + AKTi at two different time points to assess the immediate and longer term impact of the combinations in vitro. Using cell line-specific concentrations of either G12Ci or G12Di to elicit a comparable reduction in cell viability along with a single concentration of AKTi, the overall combined effect of G12Di and AKTi was synergistic in all three cell lines (CD1 < 0.9) (Fig. 5C) and significantly more potent (Supplementary Figure S8A) compared to G12Ci and AKTi at 48 h after drug exposure. At 120 h, synergy was maintained in KRAS^G12D^ lines implying that co-targeting KRAS^G12D^ and AKT is a durable treatment strategy. However, the combination became antagonistic (CDI > 1.1) for KRAS^G12C^ cell lines, suggesting that G12Ci and AKTi may be more effective separately (Fig. 5C and Supplementary S8B). Moreover, the reduction in viability with the combination of G12Di and AKTi was due to apoptotic cell death which was confirmed by rapid caspase-3/-7 activation (Supplementary Figure S9A and B). In order to confirm that the synergism between G12Di and AKTi was not due to off-target effects, we exposed KRAS^G12C^ MEFs to this combination in which no further benefit was seen compared to AKTi alone (Supplementary Figure S9C). To strengthen our findings, we next assessed the impact of co-targeting KRAS^G12D^ and mTORC1. To inhibit mTORC1, we used the third generation, bi-steric tool compound RMC-6272 which shares similar in vivo activity as clinical candidate RMC-5552 [46]. Despite not observing a genotype-specific sensitivity with mTORC1i as a monotherapy (Supplementary Figure S10A), with co-treatment alongside G12Di, we saw a synergistic impact in MEFs at 48 h (Supplementary Figure S10B) and in human lines at 120 h (Supplementary Figure S10C) compared to G12Ci and mTORC1i combination which was varied ranging from antagonistic to mildly synergistic. To further underpin the importance of inhibiting the PI3K-AKT-mTOR pathway to enhance KRAS^G12D^ inhibition, we co-inhibited either KRAS^G12C^ or KRAS^G12D^ and the MAPK pathway (ERKi). This resulted in a weakly additive response in KRAS^G12D^ cell lines and a stronger response in KRAS^G12C^ cell lines (Supplementary Figure S11A). We also assessed the G12Ci and ERKi combination in a relatively G12Ci resistant cell line (H2030- Supplementary Figure S11B) and saw similar BSS to the G12Ci sensitive H358 and HCC1171 cell lines, further indicating that targeting MAPK signaling is more effective in KRAS^G12C^ cell lines, but also suggesting that this combination is effective in both G12Ci sensitive and relatively resistant cell lines.

Following these in vitro experiments, we investigated whether the impact of co-targeting KRAS^G12D^ and AKT translated in vivo. For this, we inoculated the chorioallantoic membrane (CAM) of 10-day old chicken embryos with HCC461 or H358 cells and exposed the eggs to KRAS mutant inhibition and either ERKi or AKTi. For H358 tumors, neither AKTi or ERKi inhibition boosted the impact of G12Ci on reducing tumor weight. In fact, AKTi seemed to reduce the efficacy of G12Ci in this setting (Supplementary Figure S12A). Interestingly, in HCC461 tumors, G12Di had a greater impact on tumor weight reduction when combined with AKTi instead of ERKi (Fig. 5D), implying that this drug combination translates in vivo for human tumors. We also wanted to investigate the effect of combining G12Di with AKTi in an immunocompetent mouse model. While recapitulating many features of human NSCLC, KRAS mutant GEMMs differ in that they are unable to evoke strong anti-tumor immune responses, thus potentially lacking patient faithful responses to pharmacological intervention. Therefore, we used an immunogenic KRAS^G12D^-driven GEMM derived cell line (KPAR^G12D^) which overexpresses APOBEC3B, a single stranded DNA deaminase that induces high mutational burden and strong anti-tumor responses, resulting in a model that is overall more faithful of human disease [47]. In vitro, the combination of G12Di and AKTi resulted in a significantly stronger reduction in viability compared to the combination of G12Di and ERKi which showed no increased benefit (Supplementary Figure S12B). Interestingly, we saw that the combination of G12Di and AKTi resulted in a significantly stronger reduction in tumor volume in vivo compared to either drug alone (Fig. 5E), without affecting the health of the mice as evidenced by no obvious difference in body weights between drug-treated mice and vehicle control mice (Supplementary S12C). Altogether, these data show that rational selection of an up-front combination of KRAS mutant-specific inhibitors with inhibitors of mutant allele-specific vulnerabilities will achieve a greater therapeutic impact, minimising the risk of developing resistance. G12Di + AKTi conferred a strong therapeutic response relative to G12Di + ERKi in KRAS^G12D^ cell lines or G12Ci + AKTi in KRAS^G12C^ cell lines. Thus, in the context of KRAS^G12D^-driven NSCLC, we have identified a novel treatment vulnerability, patient selection strategy and combination approach.

## Discussion

**Fig. 6:**
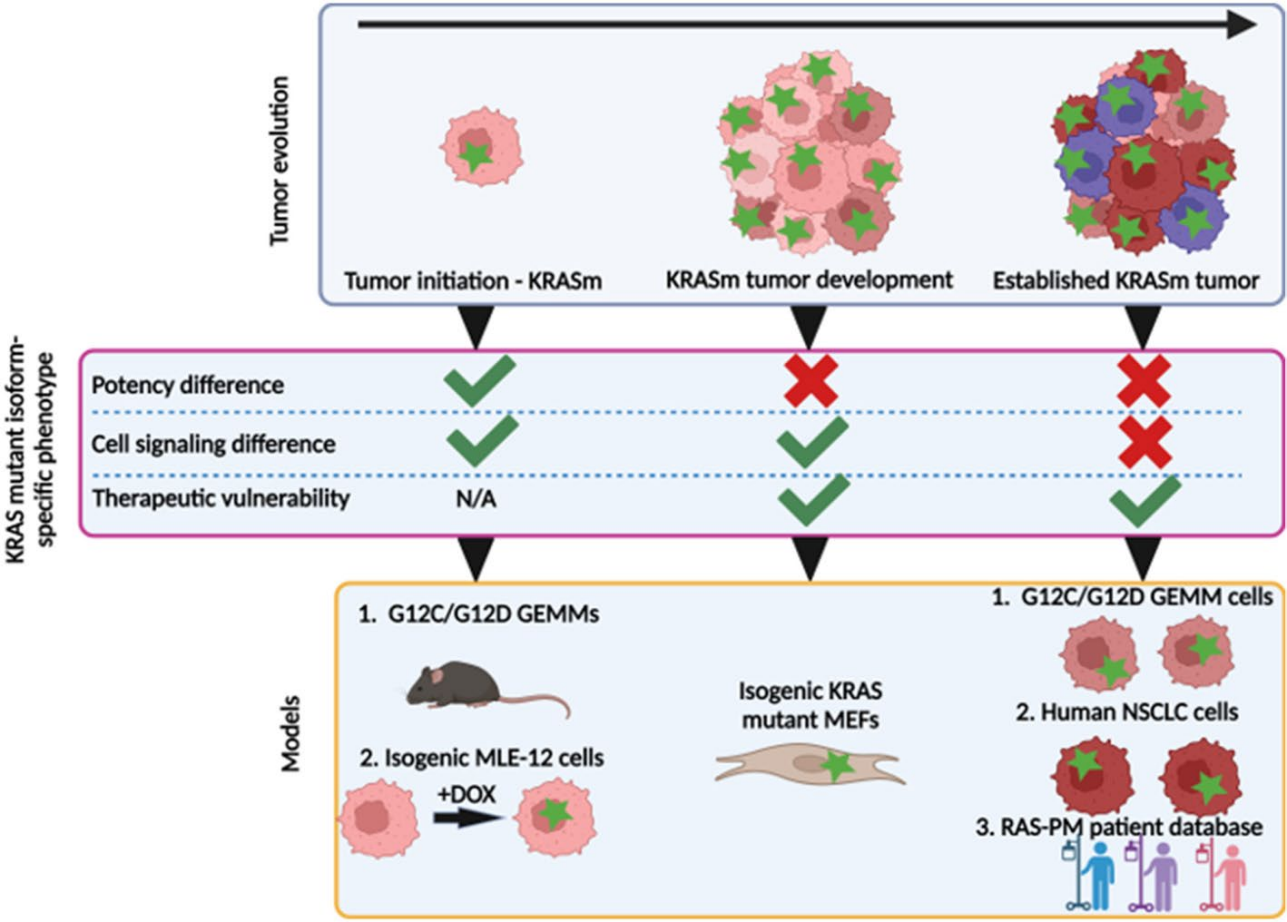
Schematic illustrating the progression of KRAS mutant-specific NSCLC. Upon initiation, the greater potency of KRAS^G12D^ induces rapid tumorigenesis relative to KRAS^G12C^ likely via the PI3K-AKT-mTOR pathway. During progression, the KRAS mutant isoform-specific differences in potency are not evident, underpinning the equivalent survival outcomes in patients. However, KRAS^G12D^ tumors maintain reliance on the PI3K-AKT-mTOR pathway, while KRAS^G12C^ increases oncogenic potential through other means, conferring therapeutic vulnerabilities which can be exploited. Green star = KRAS mutation. Created with BioRender.com/x11h794

KRAS mutant NSCLC heterogeneity limits treatment efficacy resulting in poor patient outcomes [3]. While it is becoming clear that KRAS mutations exhibit distinct biological properties [8, 9, 48], determining mutant isoform-specific treatment vulnerabilities is under-researched. Here we show that KRAS^G12D^ is more potent than the more commonly occurring KRAS^G12C^ isoform at initiating lung tumorigenesis. We also demonstrate that this superior oncogenicity may be linked to hyperactive PI3K-AKT-mTOR signaling. Interestingly, we see that this initial difference in potency is lost through tumor progression and the immediate signaling differences diminish; however, certain signaling dependencies persist or emerge during progression offering therapeutically actionable targets (Fig. 6). We propose that KRAS^G12C^ relies more on KRAS signaling through the MAPK arm to increase oncogenic potential and promote tumor growth rendering advanced KRAS^G12C^ tumors more susceptible to MAPK inhibition compared to KRAS^G12D^ tumors. In contrast, KRAS^G12D^ tumors rely on PI3K-AKT-mTOR signalling and are more vulnerable to inhibition of this pathway. Combination of KRAS^G12D^ and PI3K-AKT-mTOR pathway inhibition in KRAS^G12D^ cells was synergistic, eliciting a cytotoxic response that represents a potential mutant-specific treatment approach for NSCLC.

A striking finding from this study was the difference in lung tumor initiation and latency between mice harboring different KRAS-mutant alleles. Tumors of *Kras*^*G12D*^ mice were more abundant and grew more rapidly, translating into poorer animal survival. Interestingly, these findings mirror those from a pancreatic cancer model in which at 12 weeks after KRAS mutant activation, *Kras*^*G12D*^ mice showed more extensive pancreatic intraepithelial neoplasias (PanINs) compared to *Kras*^*G12C*^ mice. In the same study, *Kras*^*G12C*^ or *Kras*^*G12D*^ activation in colonic epithelium had an equal tumorigenic response [49]. Collectively, these reported findings and our data support the concept that the oncopotencies of KRAS mutations are tissue-specific [9], with *Kras*^*G12C*^ and *Kras*^*G12D*^ possessing contrasting potency in both the lung and pancreas, but similar potency in the colon. Our initiation model supported the in vivo phenotype, demonstrating that *Kras*^*G12D*^ is more potent than *Kras*^*G12C*^ at increasing proliferation. We also identified upregulated PI3K-AKT-mTOR signaling as a possible contributor to this more oncogenic phenotype associated with *Kras*^*G12D*^. Through inhibition of this pathway, we saw that the expression of markers associated with tumorigenesis was reduced to a greater extent in *Kras*^*G12D*^ MLE-12 cells. Furthermore, in vivo, we saw higher S6 activation in *Kras*^*G12D*^-driven early lesions. In PDAC initiation, mTOR signaling was hyperactivated in *Kras*^*G12D*^-driven acinar-to-ductal metaplasia (ADM). Genetic ablation of mTOR signaling components abolished ADM initiation [50]. Together, this report and our data both highlight that *Kras*^*G12D*^ may require input from PI3K-AKT-mTOR signaling to drive tumorigenesis compared to Kras^G12C^.

Importantly, our established tumor models and patient data reveal that in advanced disease, differences in KRAS mutant isoform-specific potency are no longer evident, mirroring findings from previous large cohort studies which failed to support KRAS-mutant allele-specific differences in outcomes [8, 51, 52]. KRAS mutant isoforms occur alongside distinct co-mutation patterns which ultimately affect signaling networks, immune surveillance and response to therapy [8, 9]. Furthermore, *KRAS*^*G12C*^ tumors have a higher mutational burden and are impacted by a higher number of co-mutations [8, 9, 19]. These genetic alterations are likely to compensate for the differences in mutant isoform-specific oncopotency. Thus, we propose that initially *KRAS*^*G12D*^ is a stronger oncogene in NSCLC and promotes rapid tumor growth compared to *KRAS*^*G12C*^. However, over time, *KRAS*^*G12C*^ tumors may acquire several additional alterations to increase tumorigenicity and, as a result, the difference in potency becomes less evident.

Our study also sheds light on the heterogeneity between KRAS mutant isoforms and isoform-specific RAS effector dependencies in established tumors. Both MEF and human NSCLC gene expression data showed enrichment of genes related to KRAS signaling (*KRAS*^*G12C*^) and PI3K-AKT-mTOR signaling (*KRAS*^*G12D*^). Interestingly, this did not align with phosphorylation levels of effector proteins within these pathways in our panel of NSCLC cell lines as they exhibited heterogeneous phosphorylation levels that were cell line-specific, rather than KRAS mutant isoform-specific. Surprisingly, despite the varied phosphorylation levels, we saw KRAS mutant isoform-specific responses to MAPK or PI3K-AKT-mTOR inhibition in NSCLC cell lines. We observed that ERK and MEK inhibition individually had a stronger impact on viability in *KRAS*^*G12C*^ NSCLC lines compared to *KRAS*^*G12D*^. This is in agreement with a previous study using a MEK inhibitor in an isogenic MEF panel, which reported greater sensitivity of Kras^G12C^ MEFs to MEK inhibition despite exhibiting comparable levels of phosphorylated MEK with Kras^G12D^ MEFs [8]. Additionally, *KRAS*^*G12D*^ NSCLC cell lines were more susceptible to PI3K-AKT-mTOR inhibition. The varied levels of PI3K-AKT-mTOR activation observed among NSCLC cell lines may be influenced by factors such as different levels of KRAS expression or co-mutations. However, despite the heterogeneity in *KRAS*^*G12D*^ cell lines, this pathway still acts as a critical node to maintain tumor viability. Clinical trials have returned disappointing results for agents targeting PI3K-AKT-mTOR signaling as monotherapy in NSCLC. However, these trials were carried out on molecularly unselected cohorts [53]. Despite AKT activating mutations being rare, AKT isoforms are overexpressed in NSCLC [54] and there are reports of efficacy with AKT inhibition as part of combination treatments in lung cancer patients [55], implying that there is potential for NSCLC patients to benefit from PI3K-AKT-mTOR targeted therapy. In light of this and our findings, we propose targeting PI3K-AKT-mTOR signaling in *KRAS*^*G12D*^-driven LUAD as a potential therapeutic option. Additionally, as our MEF and publicly available human CCLE gene expression data showed similar gene-set enrichment which informed treatment vulnerabilities, it is worth considering that genotype-specific transcriptional signatures are better determinants of therapeutic vulnerabilities rather than pathway activation.

As already mentioned, studies have shown that KRAS mutant isoforms exhibit distinct tissue-specific features. Thus, dissecting the individual functions of KRAS mutant isoforms in the context of NSCLC is critical to inform combination partners to maximise the effectiveness of direct KRAS inhibitors in this disease setting. There are emerging reports of effective combination treatments to increase the effectiveness of KRAS^G12D^ inhibition in colorectal cancer [42, 43]. However, to our knowledge, there has not been any investigation into combinatorial strategies involving KRAS^G12D^ inhibition in NSCLC. We showed that the combination of KRAS^G12D^ inhibition and PI3K-AKT-mTOR pathway inhibition co-operated effectively in reducing tumor viability in vitro and in vivo, whilst other combinations were mostly additive or antagonistic.

To conclude, our study emphasises that different KRAS mutations exhibit different oncopotencies which become less apparent over time despite retaining intrinsic dependencies resulting in therapeutic vulnerabilities. More specifically, it highlights that one amino acid difference between RAS point mutations dictates precision medicine approaches in NSCLC. Our data supports the idea that solely confirming the presence of KRAS mutation in a patient with NSCLC is insufficient to inform treatment options. Rather, knowing the type of KRAS mutation is critical especially now with the new wave of KRAS mutant isoform-specific inhibitors emerging which require combinatorial treatments to maximise therapeutic efficacy and decrease resistance.

## Supplementary Information

Supplementary Material 1.

## Data Availability

RNA-seq data can be accessed within the BioProject database (NCBI) using ID number PRJNA1034560.

## Abbreviations

4E-BP1: Eukaryotic translation initiation factor 4E binding protein 1
AdCre: Cre-expressing adenovirus
BSS: Bliss Synergy Score
CAM: Chorioallantoic membrane
CCLE: Cancer Cell Line Encyclopedia
CDI: Coefficient of drug interaction
DEG: Differentially expressed gene
DOX: Doxycyline
ERK 1/2: Extra-cellular Signal Regulated Kinases 1 and 2
GEMM: Genetically engineered mouse model
KRAS: Kirsten rat sarcoma viral oncogene homologue
LUAD: Lung adenocarcinoma
MAPK: Mitogen-activated protein kinase
MEF: Murine embryonic fibroblast
MEK: Mitogen-activated protein kinase
MLE-12: Murine lung epithelial
mTCL: Murine tumor cell line
mTOR: Mammalian target of rapamycin
mTORC1: Mammalian target of rapamycin complex I
NSCLC: Non-small cell lung cancer
OS: Overall survival
RAS-PM: RAS precision medicine
PFS: Progression-free survival
PFU: Plaque-forming units
PI3K: Phosphatidyl inositol 3-kinase
TCGA: The Cancer Genome Atlas
ULA: Ultra-low attachment
WT: Wildtype
